# Spontaneous production rates in music and speech: Effector systems or domain specificity?

**DOI:** 10.3389/fnhum.2026.1744449

**Published:** 2026-02-26

**Authors:** Nicole C. Coleman, Caroline Palmer, Peter Q. Pfordrsher

**Affiliations:** 1 Department of Psychology, University at Buffalo, Buffalo, NY, United States; 2 Department of Psychology, McGill University, Montreal, QC, Canada

**Keywords:** domain specificity, music, singing, speech, spontaneous production rates

## Abstract

Individuals perform many tasks at an optimal rate that is consistent within but not between individuals, evidenced by the spontaneous rate at which one performs a task in the absence of external rate cues. We tested three hypotheses concerning how spontaneous production rates (SPRs) are generated and associated across language and music tasks: Biomechanical constraints associated with effector systems (vocalized/fingered: H1), reliance on auditory feedback (presence/absence: H2), and domain-specific constraints (speech/music: H3). We tested these hypotheses by having participants produce music and speech sequences, sequences that used vocalized or fingered effectors, plus a Silent finger-tapping condition to test the influence of auditory feedback on spontaneous production rates. SPRs were significantly correlated across all tasks that involved production of auditory feedback, regardless of effector or domain. However, Silent Tapping rates were not significantly correlated with any SPRs that produced auditory feedback. Together, these findings suggest that the generation of auditory feedback plays a critical role in the spontaneous rate at which participants engage in rhythmic motor actions, more so than the biomechanical constraints of effector systems.

## Introduction

People engage in a variety of rhythmic motor behaviors, including walking and running, which are defined in part by a regular period or rate at which the rhythm recurs. A wide range of movement rates are available, but the range is always limited and in general there is an optimal rate that an individual gravitates toward for any given behavior. These optimal rates are estimated using the *spontaneous production rate* (SPR), which is the rate at which one produces sequences of sounds in the absence of external cues. We address rhythmic behaviors that are used to communicate via audition: music and speech. These behaviors are associated with different cognitive *domains*, referring to a class of information that may be associated with a distinct cognitive network, possibly associated with distinct modules (Fodor, 1983). Music and language may constitute separate domains according to some claims (e.g., Patel, 2008; Peretz and Coltheart, 2003). With respect to the rate at which people produce music and speech, two questions emerge: First, is there any association between an individual’s optimal rate for speech and their optimal rate for music production, and—if so—the second question involves the basis on which that association is made.

The second question is of particular interest given that the production of music and language can vary both in terms of the actions used to generate *auditory feedback*, and in terms of the *effector system* used to produce the actions. We define effector systems as a collection of muscles and joints, such as finger or tongue movements, that function in a coordinated way in order to execute the goals of an action, forming synergies (Bernstein, 1967). The present study addresses the role of these factors in the Spontaneous Production rates (SPRs) at which people produce rhythmic sequences like speech or music.

We focus primarily on how individual differences are associated across music and speech production tasks, and not on the average rates at which each task is produced. Recent evidence suggests that music and language from around the world differ with respect to the rate at which they are produced, with considerably faster rates for the rhythms formed by successive spoken syllables than by the onsets of musical notes in a melody (Ding et al., 2017; Ozaki et al., 2024). The fact that these differences exist does not necessarily indicate that different timing systems contribute to an individual’s optimal rates of production across domains. It is well known that different gait patterns within species, including horses (Hoyt and Taylor, 1981) and humans (Kung et al., 2018) are associated with different optimal rates, for example with faster optimal rates for running and slower optimal rates for walking. These differences can be attributed to changes in the attractor landscape within a single system when a control pattern (here, rate) varies, and do not require any attribution to the use of different timing systems for the different coordination patterns (e.g., Kelso, 1995). In a similar way, it is possible that SPRs for speech and music production may vary in overall rate while being based on the same timing system. Given this flexibility, we focus on whether an individual who chooses a relatively “fast” rate for one task (compared to other individuals) also chooses a “fast” rate on a different task, even if the two rates differ considerably from each other. If so, we may say that this individual timekeeper engages a “fast” timekeeper during both motor tasks, while varying rates flexibly from instance to instance in response to other contextual factors.

Results from previous studies offer mixed evidence concerning whether SPRs are correlated across music and language production. Whereas Pfordresher et al. (2021) found no significant correlation between SPRs for speech production and the production of melodies on a keyboard, Coleman et al. (2024) found significant associations between production of speech and production of sung melodies. These results are consistent with the view that SPRs are based on the effector system used, given that the correlated SPRs in Coleman et al. were found when speech and music were produced via the same system. Similarly, Engler et al. (2024) found that tapping rates were more strongly correlated when participants tapped under conditions of varying auditory feedback (taps producing a melody, a single tone, or silent) in contrast to correlations between tapping rates with either hand clapping or walking. Constraints on optimal rates due to effector system may reflect the biomechanics associated with effector systems that function synergistically when one engages in rhythmic movements. The integrated movements of limbs that vary in mass, flexibility and muscular dexterity significantly influence the optimal rate of rhythmic movements. These effects have been modeled in past research by varying masses of pendula that are moved rhythmically (e.g., Turvey et al., 1986). These studies did not, however, manipulate the presence or absence of auditory feedback, an important dimension of timing in music and speech.

Other results suggest that auditory feedback plays a role in governing optimal rates. Tranchant et al. (2022) found that the spontaneous rate of each participant’s productions of musical sequences using finger movements were not significantly correlated with their spontaneous motor tempo (fingered tapping at consistent rates in the absence of auditory feedback; Drake et al., 2000; McAuley et al., 2006), whereas other studies have found significant correlations for similar conditions (Engler et al., 2024; Pfordresher et al., 2021). Engler et al. (2024) found stronger correlations between tasks with shared auditory information—tapping to produce different melodies, or rhythmic actions that generated no sound (silent tapping versus hand clapping)—than across tasks that generate different kinds of auditory patterns. These results suggest that auditory information associated with produced actions—here the simple presence or absence of auditory feedback—may determine optimal production rates.

Other theoretical approaches associate spontaneous production rates with information based on principles of information transmission, possibly leading to differences in production rates across music and speech (Assaneo and Poeppel, 2018; Doelling et al., 2019). Thus, differences in SPRs may result for actions that lead to music or speech, independent of effector system.

The present study tests three hypotheses concerning the basis on which timekeepers may or may not be distinguished. The first (H1) is that spontaneous rates are constrained by the biomechanics of the effector system used to generate a rhythm. Some aforementioned results support H1: Whereas Coleman et al. (2024) found significantly correlated SPRs across speaking and singing, which share the same (vocal) effector system, Pfordresher et al. (2021) found no significant association (and a near zero correlation) for SPRs from speaking and playing melodies on a piano. The current study tests whether this dissociation is replicated within a single study.

The second hypothesis (H2) is that SPRs are influenced by the presence of auditory feedback generated from actions. Recent studies provide mixed evidence. Whereas Tranchant et al. (2022) found no association between silent tapping and a condition in which tapping led to a sequence of melody pitches, Pfordresher et al. (2021) found a significant but modest correlation between silent tapping and SPRs for piano production. As shown in Table 1, H2 predicts strong correlations between spontaneous rates among sung melodies, speech, and tapped melodies, for which feedback is present, but weak or non-significant correlations between any of these conditions and silent tapping.

**Table 1:** Research hypotheses and conditions.

Condition	Effector (H1)	Auditory feedback (H2)	Domain (H3)
Speech	Vocal	Present	Language
Sung Melody	Vocal	Present	Music
Tapped Melody	Fingered	Present	Music
Silent Tapping	Fingered	Absent	n/a

Columns after experimental conditions organized by factors that are related to the three hypotheses being tested. ‘Effector’ refers to the effector system used for a task, relevant to hypothesis H1. ‘Auditory Feedback’ refers to the presence or absence of auditory feedback, relevant to hypothesis H2. Domain refers to whether the auditory information conforms to the domain of Language or Music. Tests of hypotheses are based on whether correlations of production rates are based on one or more of these factors.

The third hypothesis (H3) is that SPRs are based on the acoustic patterning of domain-specific information that is produced. This hypothesis predicts that SPRs will be correlated within domain (i.e., across production of music via singing and on a keyboard), but not across domains (i.e., music versus speech production). Although the results of Pfordresher et al. (2021) are consistent with H3, the results of Coleman et al. (2024) are not. However, neither of these studies included comparisons within a domain across different effector systems. A recent study that examined synchronization that crossed effector system (hand claps versus articulation) with the domain associated with the auditory target (syllable versus tone), was consistent with the idea that timekeeping is independent of effector and domain for accurate synchronizers (Mares et al., 2023). The current study uses a comparable design and can address whether this generality extends to SPRs.

We report an experiment that tested these hypotheses by having participants generate SPRs for short memorized sequences. Participants included those with and without musical training. Tasks that could be produced easily (without formal training) were presented to each participant in four conditions: Speaking short phrases, singing familiar melodies on the syllable ‘da’ (Sung Melody), producing familiar melodies by tapping on a keyboard (Tapped Melody), or Silent Tapping. Participants were given different melodies for the Sung Melody and Tapped Melody conditions to avoid carryover effects across conditions. Table 1 illustrates the relationship between these experimental conditions and the three hypotheses. Whereas Speech and Sung Melody conditions both involve the vocal effector system, Tapped Melody and Silent Tapping conditions both involve the hand and finger effector system. H1 predicts shared SPRs within the verbal and within the tapping conditions, but not across these systems. The Silent Tapping condition was used to test H2: If the presence of auditory feedback determines SPRs, then production rates in this condition should not correlate with the Tapped Melody condition. H3 predicts a significant association between Sung and Tapped Melody conditions, but no correlation between these conditions and the Speech condition.

## Method

### Participants

Eighty-one students were recruited through the University at Buffalo’s online participant recruiting software (SONA) and given partial course credit for Introduction to Psychology in exchange for their time. Participants gave informed consent to participate in the study and indicated they had not been diagnosed with any hearing or speaking disabilities. Twenty-three participants were excluded because of unfamiliarity with the melodic stimuli (*n* = 20) or errors/dysfluencies during production of speech stimuli (*n* = 3).

Fifty-eight participants (mean age = 19.07, range = 18–29) were included in the final analysis. These participants had on average 0.66 years (range = 0–16 years) of formal training in singing (e.g., private lessons) and 2.91 years (range = 0–17) in organized singing (e.g., choir). Participants also averaged 2.85 years (range = 0–15 years) of formal training on a primary instrument. Six participants (10% of the sample) were non-L1 English speakers. Two participants listed Chinese (including Cantonese, Mandarin, and other Chinese languages) and two participants listed Korean as their native language. Two participants listed ‘other’ without specifying their native language. Of the individuals who were L1 English speakers, 28 (48% of the sample) were multilingual.

### Apparatus

Participants were seated in front of a computer monitor (Dell ST2310F) and a M-Audio Keystation 49e keyboard. The computer monitor was connected to and mirrored the screen of a windows computer (Dell OptiPlex 7490 AIO) outside of the sound booth. The windows computer ran the Speech and Sung Melody tasks through MATLAB (version R2021b) using Psychtoolbox-3 (Kleiner et al., 2007). A three-key mini keypad in the booth allowed the participants to progress through the Speech and Sung Melody tasks at their own pace. Participants spoke or sang into a microphone (Shure SM58). Audio for these tasks was recorded through an audio interface (Focusrite Scarlett 2i2).

Participants tapped on the M-Audio Keystation 49e, using a single key (see Procedure) for the melody tapping and Silent Tapping trials. Each tap generated the next tone in the melody, allowing musically trained and untrained participants to control the melody rate. Melodies were played through a Roland RD-700 SX midi tone generator over Sennheiser HD 280 pro (64 ohm) headphones. FTAP, a software package used to collect MIDI data and control MIDI output with millisecond temporal precision (Finney, 2001), was used to output the experimental orders and acquire tapping responses. FTAP was run on a Linux operating system on a Dell Optiplex 790 computer outside of the booth.

A microphone (AKG C1000S) outside of the booth allowed experimenters to talk to the participant and a pair of headphones (Sennheiser HD 280 pro) allowed the experimenter to hear the participant. Qualtrics (Provo, UT, USA) was used to administer the questionnaire at the end of the experiment.

### Materials and design

There were four spontaneous rate tasks: Speaking, singing (Sung Melody), production of melodies by tapping (Tapped Melody), and tapping without auditory feedback (Silent Tapping). These conditions varied with respect to effector system used for production (vocal: Speech and Sung Melody, fingers: Tapped Melody and Silent Tapping), presence of auditory feedback (absent for Silent Tapping, present for the rest), and—for conditions with auditory feedback—domain represented by auditory information (speech versus music). Participants produced two stimulus items for each task generating auditory feedback and completed three successive trials for each stimulus item. Tasks were grouped together in blocks to avoid confusion for participants.

**Figure 1:**
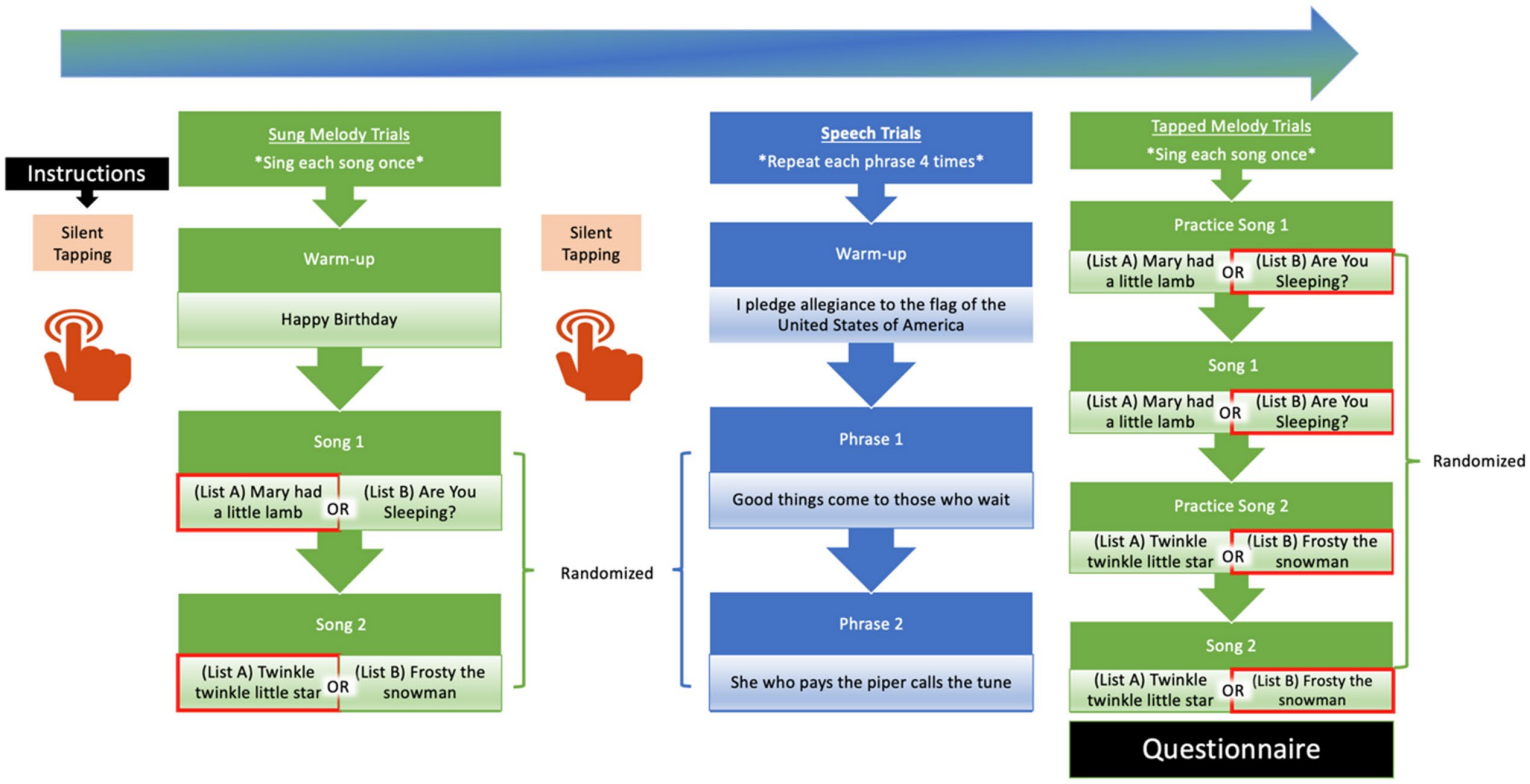
Illustration of trial orders. This is an example depicting Order 1 for a given participant. There are 6 trials total for each Speech or Sung Melody condition and each condition began with practice trials. For the music tasks (Sung and Tapped Melody), four melodies were assigned. Two of the melodies were produced in the Sung Melody trials and the remaining melodies were produced in the Tapped Melody trials, as indicated by the red outlines for each stimulus.

Each participant was randomly assigned to one of two experimental orders for the succession of tasks. Both orders followed two constraints. First, musical production tasks (Sung and Tapped Melody tasks) did not follow each other in direct succession to minimize potential within-domain carryover effects. Second, each order began with a Silent Tapping trial to provide an estimate for this task that was free of any possible carryover of auditory information from previous tasks. Order 1 was Silent Tapping, Sung Melody Trials, Silent Tapping, Speech Trials, and Tapped Melody Trials. Order 2 was Silent Tapping, Tapped Melody Trials, Speech Trials, Silent Tapping, and Sung Melody Trials. Figure 1 illustrates the flow of trials for an example participant in Order 1.

During the Speech task, two short phrases served as stimulus items. Participants were asked to memorize all two phrases at the start of the task. The phrases were: *Good things come to those who wait*, and *She who pays the piper calls the tune*. These phrases were chosen because they are simple, have a binary meter, are simple in structure, and the stressed syllables are easy to distinguish when marking onsets during analysis (see *Data Analysis*).

For the Sung Melody task, participants were asked to produce two familiar melodies from either list A or list B from memory: *Mary Had a Little Lamb* and *Twinkle Twinkle Little Star (List A)* or *Frosty the Snowman* and *Are You Sleeping? (List B)*, to ensure that the same melodies were not used for both sung and Tapped Melody trials for a given participant. List order was counterbalanced with experimental order (Order 1 and Order 2). Participants only had to be familiar with the tune and not the song lyrics; all participants sang the melodies on the syllable “da.” The songs were chosen because they are highly familiar to most participants and have a binary meter. Additionally, these songs are not associated with a canonical pre-recorded version and thus will not be associated with a specific tempo (as would be the case for prerecorded popular music) to avoid effects related to tempo recall (e.g., Hine et al., 2025). If they were unfamiliar with a melody, they were asked to choose an alternate familiar melody from the same list for the remaining three trials.

For the Tapped Melody task, participants heard the melody they had chosen as familiar from list A or B. On each trial, participants tapped a single key on the keyboard and each tap generated the next pitch in the melody, based on MIDI pitch information presented by the FTAP software package (Finney, 2001). Successive presses of the key thus allowed the participant to control the melody’s rate. Participants practiced tapping the melody in a practice trial until they felt comfortable to move on to the experimental trials.

For the Silent Tapping task, participants were instructed to tap on a single key on the keyboard in front of them at a comfortable and consistent rate until instructed by the experimenter to stop. The audio output from the keyboard was turned off, and no keyboard sounds resulted from their actions. This task occurred before and after the first block of trials and consisted of 39 taps on each trial.

A background questionnaire measured participants’ ages at which they started learning any non-native languages as well as their musical background, using self-reported years of training in organized singing (including choir, musical ensembles, musical theater, etc.), formal training in singing, and formal training on any primary instrument. We screened for the possibility of congenital amusia, a disorder affecting the perception of pitch and overall recognition of music (Ayotte et al., 2002; Peretz and Vuvan, 2017), by asking the level of difficulty a participant had in recognizing music when lyrics are absent, and we screened for possible beat-deafness (Phillips-Silver et al., 2011) by asking participants how hard it was for them to find the beat of the music.

### Procedure

Participants were directed to enter the sound-attenuated booth. Once the participant read and provided informed consent, the experimenter sat down at the desk outside of the sound booth to start the experiment. The participant and experimenter communicated through the microphone and headphone set-up on both sides of the sound booth. The experimenter then guided the participant through the correct order of tasks, reading the instructions for each task and asking if the participant had any questions along the way. In each task, participants were instructed to produce the taps or vocalizations at a consistent and comfortable tempo. None of the participants had difficulty understanding or complying with this general instruction, monitored by an experimenter.

Each order began with the Silent Tapping condition. Participants were instructed to tap at a comfortable and consistent rate using the pointer finger of their dominant hand until the experimenter instructed them to stop. Although no sound was played through the headphones, participants were instructed to keep the headphones on to minimize any sound occurring from any keyboard movement. The participant completed one trial of the Silent Tapping task before moving on to the next task.

Using Order1 as an example, participants next began the Sung Melody trials by producing a warm-up melody, *Happy Birthday*. At the beginning of each Sung Melody trial, song lyrics were displayed on the screen to aid participants’ memory for the melody until the participant decided to progress to the production task at which point the lyrics were removed. The participant was then instructed to repeat the melody at a comfortable and consistent rate on the syllable “da” to reduce memory demands, provide a clear consonant sound to mark syllable onsets, and prevent the length of the phonemes from influencing their spontaneous singing rate, (e.g., Racette and Peretz, 2007). After completing one warm-up trial, the participant completed three consecutive trials for each of the two chosen melodies, which were presented in a randomized order.

The participant then repeated another trial of the Silent Tapping and was told to tap at a comfortable and consistent rate until instructed to stop by the experimenter.

Next, the participant completed a block of Speech trials. A single *trial* for speech was defined as 4 successive repetitions of a short well-known phrase. Four repetitions of each spoken phrase in the Speech trials approximately matched the duration of the Sung Melody trials. On each trial the participant viewed the text of the target phrase on the computer screen. They were instructed to read and memorize the phrase on the screen and then proceed to the recording screen when they were ready, at which time the phrase disappeared from the screen. They spoke the phrase from memory to avoid the effects of reading on spontaneous speaking rate and to encourage a more natural speaking rate. The participant was instructed to repeat the phrase at a consistent and comfortable rate from memory. If the participant needed more time to see or practice the stimulus, the phrase appeared again on the screen until the participant was ready to record. The block of Speech trials began with a warm-up trial that involved producing from memory *I pledge allegiance to the flag of the United States of America* four times, as shown in Figure 1. The warm-up trial, similar to the Happy Birthday Sung Melody warmup, helped to ensure that participants understood the instructions and performed the tasks correctly. There were three consecutive trials for each phrase in the Speech task using the target phrases described earlier.

The participant then proceeded to the Tapped Melody task. This block of trials was configured identically to the Sung Melody trials, only using the alternate list of melodies (i.e., if Sung Melody trials used list A, then Tapped Melody trials would use list B). Participants produced the melodies by tapping a single key on the keyboard. Participants were instructed to produce the melody at a comfortable and consistent rate. Warm-up for this task involved tapping a practice melody until the participant tapped the rhythm correctly.

At the end of the experiment, the participant was instructed to sit at the windows computer outside of the sound booth where the questionnaire appeared on the screen. The participant then completed the background questionnaire. When the questionnaire was submitted, the researcher granted course credit to the participant and ended the experiment session. The entire experiment lasted 60 min.

### Data analysis

Participants’ SPRs were assessed by marking sung tone onsets and syllable onsets and by calculating the mean of all inter-onset intervals (IOIs) for each trial (one melody repetition for music; four repetitions of one spoken phrase for speech). For speech production, the onset of each syllable was identified using Gentle (Version 0.10.01), a forced aligner program that uses media files and audio transcripts to mark the onset of each phoneme automatically and precisely. Analyses of vocal and fingered melodies omitted the IOI defined by the onset of the final syllable of a phrase and the onset of the first syllable of the next phrase in a trial because participants tended to pause in-between repetitions (cf. Pfordresher et al., 2021). The inclusion of these pauses would inaccurately inflate an individual’s SPR.

For Sung Melody production, the onset of each “da” tone was marked manually using Praat (Boersma and Weenink, 2022). The researcher used visual cues from the spectral content and amplitude envelope of the sound in addition to auditory cues to hand-mark the onset of each ‘Da’. The denominational unit we used to establish SPR was the quarter note. For Sung Melody and Tapped Melody trials, IOIs that included denominations of half notes or longer were omitted because participants tend to shorten long durations, making the ratio of half notes to quarter notes closer to 1:1.7 rather than the correct notated ratio of 1:2 (Bengtsson and Gabrielsson, 1980; Repp et al., 2013). Likewise, we summed across IOIs for 8th notes that followed in immediate succession, so that the resulting measure mapped onto the quarter note duration. We removed 8th note IOIs that were isolated (e.g., the 8th note that follows the dotted quarter note in *Frosty the Snowman*). For trials on which the participant misinterpreted the rhythm (which occurred on 17% of all Sung Melody trials, but not on any Tapped Melody trials), we created a template of notated durations based on the rhythm produced by the participant, rather than the ideal notated rhythm for the melody. After these adjustments, all IOIs in the remaining trials were used in the Sung Melody analysis as participants did not add additional pauses in between notes or phrases. The timing data through FTAP for the Tapped Melody trials and the Silent Tapping trials provided the information to calculate the IOI for each tap. No IOIs were excluded from the Silent Tapping tasks as the participants did not pause during their productions.

The mean IOI was first calculated for each trial, following removal of outliers. The mean and SE were then computed across the trial means and constituted the primary dependent variable. Outliers were defined separately for each participant and task (Speech or Sung Melody) as being more than three standard deviations away from the mean across all trials in the IOIs used to analyze a single trial. This process led to negligible loss of data (*N* = 55 IOIs removed, 67 from Speech trials and 8 from Sung Melody trials, 0.002% of all IOIs). Any dysfluencies were removed in the process of removing outliers.

## Results

**Figure 2:**
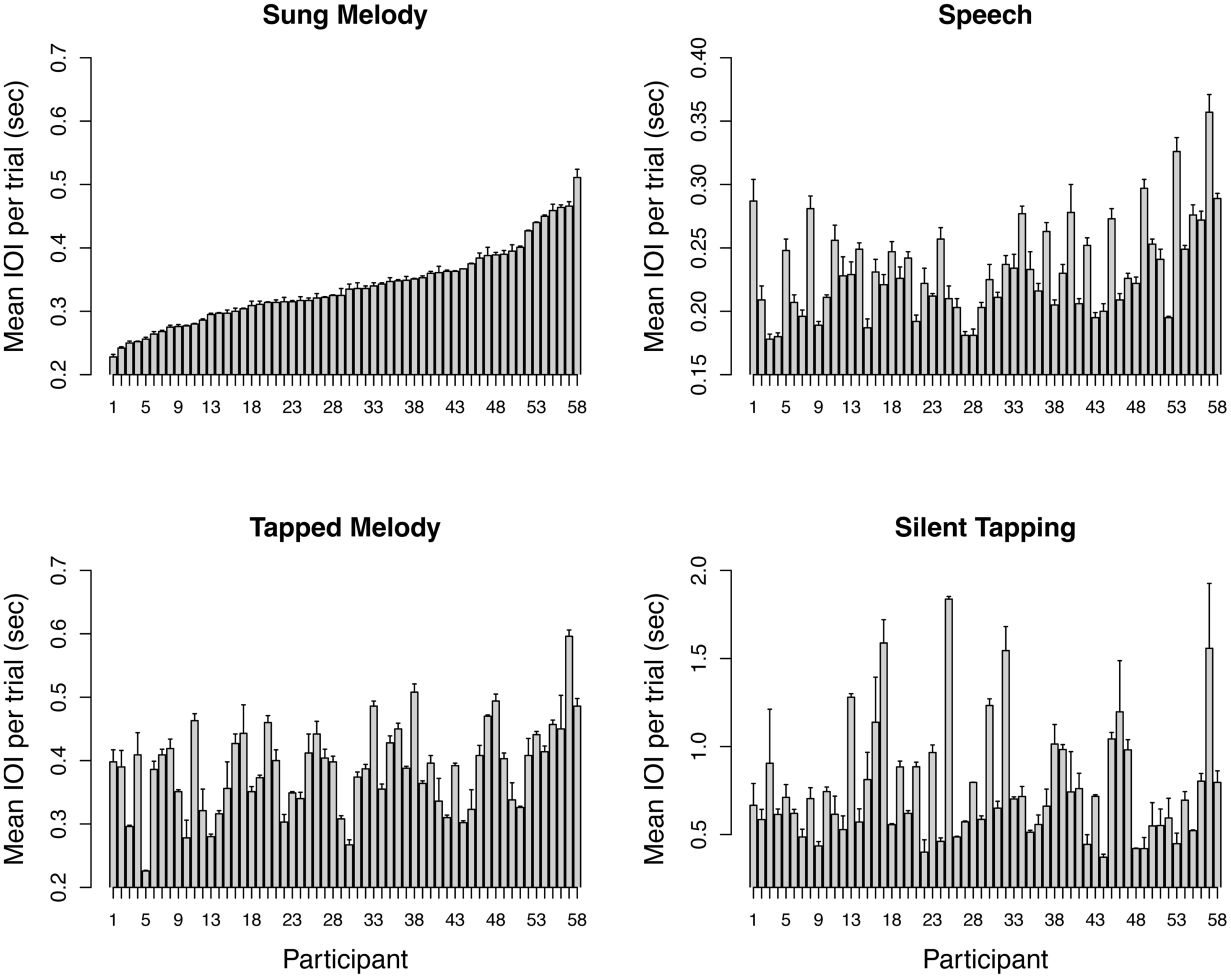
Mean IOI by participant for each task. Mean (SE) SPR across item using mean IOI for each individual for all 4 production tasks. Participants are ordered on the *x*-axis in all graphs based on the Sung Melody task. *Y*-axis scale varies across graphs to highlight the range of individual differences within each task. Mean and SE values are based on the mean across trials for each task (*n* = 6 for Sung Melody, Speech, and Tapped Melody, *n* = 2 for Silent Tapping).

Figure 2 show the distribution of mean IOIs across participants. Bars in each panel represent the mean IOI across trials, and error bars reflect variability across trials for that participant and task. Participants are ordered in each panel according to their rank order for Sung Melody IOIs, and axis scales vary to show the range of IOIs in the task (e.g., note that smaller *Y*-axis range for Sung Melody trials than music trials). These plots verify that individuals varied significantly from each other on each task, thus validating the use of correlation to assess the degree of covariation across tasks.

**Figure 3:**
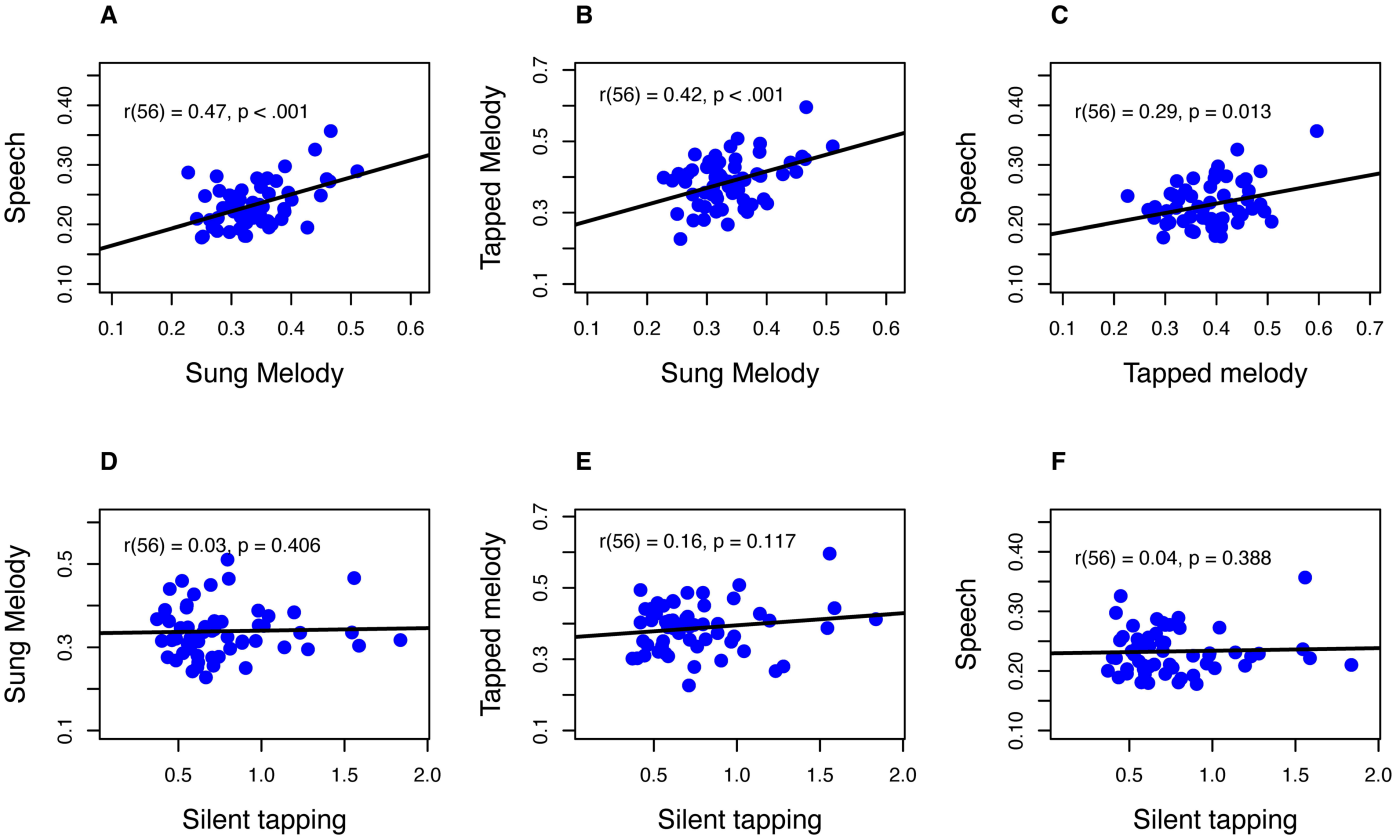
Bivariate correlations across tasks. All axis units are in seconds per IOI, on average. Axis scales vary by panel based on the range of X and Y values. Panels **(A–C)** (top row) show correlations among SPR tasks that include auditory feedback. Panels **(D–F)** (bottom row) show correlations of SPR tasks with Silent Tapping.

Figure 3 displays associations between SPRs across tasks. Each point in each plot represents the joint mean SPR for two conditions. Because each condition participates in three different bivariate correlations, we adopted a Bonferroni correction and considered correlations at *p* < 0.017 to be statistically significant. Pearson correlations are shown in the leftmost column of Table 2. All correlations that compared conditions in which actions led to auditory feedback (SPR tasks), yielded significant correlations. By contrast, all correlations with Silent Tapping (bottom row of Figure 3) did not yield significant correlations (*p* > 0.10 for each).

**Table 2:** Pearson *r* and Bayes Factor values for associations across tasks.

Correlation	Pearson *r*	Bayes Factor_10_	Bayes Factor _01_
Sung Melody/Speech	0.47**	279.46+++	0.004
Sung Melody/Tapped Melody	0.42**	59.938++	0.017
Speech/Tapped Melody	0.29*	3.558+	0.281
Sung Melody/Silent Tapping	0.03	0.137	7.278−−
Tapped Melody/Silent Tapping	0.16	0.123	8.120−−
Speech/Silent Tapping	0.04	0.119	8.408−−

All tests one-tailed based on anticipated positive association. Bayse Factor_10_ assesses support for alternative over null hypothesis, whereas Bayes Factor_01_ assesses support for null over alternative hypothesis. For Pearson *r*: **p < 0.001, *p < 0.017. For Bayes Factor_10_: +++ extreme evidence, ++ very strong evidence, + moderate. For Bayes Factor_01_: −− very strong evidence.

We also analyzed the results shown in Figure 3 using the Bayes Factor criterion (Rouder et al., 2009). Using the Bayesian correlation function in JASP version 0.17.2.1 (JASP Team, 2023), the Bayes Factor criterion (BF) tests the likelihood of the alternative hypothesis (significant correlation, BF_10_) over the null (no correlation) or support for the null over the alternative (BF_01_). Because only positive associations of SPRs across tasks are interpretable, we treated all tests as one-tailed. BF values, shown in the middle and right columns of Table 2, indicated overwhelming support of the alternative hypothesis (i.e., a significant correlation) for correlations of Sung Melody with both Tapped Melody and Speech, and the association between Speech and Tapped Melody SPRs yielded strong support for the alternative hypothesis. By contrast, all associations involving Silent Taping yielded strong support for the null hypothesis. These results were consistent with those found for frequentist (Pearson) correlations, while providing additional insights on whether results support the null hypothesis.

We also analyzed whether this pattern of results differed by language experience or musical background. The current sample included a sizable proportion of multilingual participants and included predominantly nonmusicians. Although the vast majority of multilinguals in our sample were L1 English speakers, it is possible that experience learning the sound patterns of other languages may influence speech SPRs. Table 3 shows the across-task correlations for all participants in comparison with multilingual and monolingual participants. The same basic pattern is found in all groups, although the correlation between Tapped Melodies and Speech (more modest in size for the entire group than the other significant correlations) was non-significant for the two smaller subsets (despite being of higher magnitude for multilingual participants than for the whole sample). We take this to be simply a byproduct of sample size. We also analyzed correlations for those participants who were categorized as non-musicians, defined as having fewer than 6 years of formal training on any musical instrument or singing. These correlations also yielded the same pattern as found for the entire sample. We did not run correlations on participants defined as musicians given how few participants were categorized as such (*n* = 10).

**Table 3:** Pearson *r* values for participant groups.

Correlation	Multilingual (*n* = 20)	Monolingual (*n* = 38)	Non-musicians (*n* = 48)
Sung Melody/Speech	**0.55**	**0.39**	**0.54**
Sung Melody/Tapped Melody	**0.49**	**0.36**	**0.45**
Speech/Tapped Melody	0.37	0.23	**0.31**
Sung Melody/Silent Tapping	−0.03	0.05	0.03
Tapped Melody/Silent Tapping	0.02	0.21	0.11
Speech/Silent Tapping	−0.03	0.06	0.03

Bold indicates correlations significant at *p* < 0.017.

We analyzed correlations of the Silent Tapping condition with other conditions based on its relative position within the order of tasks. One complication with interpreting this condition as representing the absence of feedback is that in certain situations participants may imagine auditory feedback resulting from their taps (Brown and Palmer, 2012, 2013; Highben and Palmer, 2004). This is particularly likely for participants who experience the second silent tapping trial after the Melody Tapping condition, which is similar to Silent Tapping with respect to the effector system. This happens in the second of two orders that a participant could experience (see *Methods*). Thus, we re-analyzed all correlations of Silent Tapping with the other conditions, separately for the first and second trials of Silent Tapping (trial 1 always began the session), and further separating correlations with trial 2 for participants who experienced trial Order 1 (Silent Tapping precedes Melody Tapping) versus those who experienced trial Order 2 (Silent Tapping follows Melody Tapping). Spontaneous tapping rates for the second trial of Silent Tapping did correlate significantly with Melody Tapping for participants who experienced trial Order 2, *r*(27) = 0.35, *p* = 0.03, suggesting that auditory imagery associated with Melody Tapping may have carried over to the later Silent Tapping trials in these cases. No other correlations of any SPR trials with Silent Tapping were significant (all *p* > 0.10).

**Figure 4:**
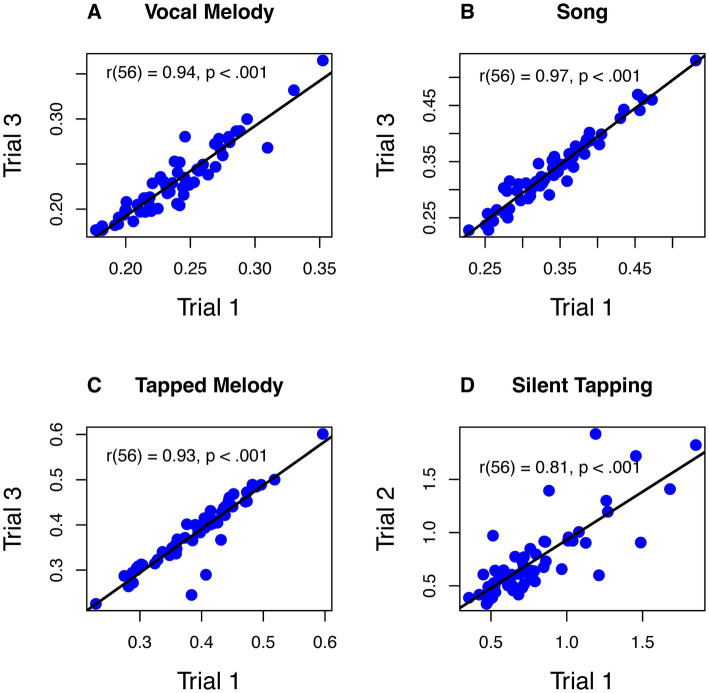
Bivariate correlations across trials and within tasks (**A** = Vocal Melody, **B** = Song, **C** = Tapped Melody, **D** = Silent Tapping). All axis units are in seconds per IOI, on average. Axis scales vary by panel based on the range of X and Y values.

We next addressed whether SPRs are reliable across trials within a given task (Figure 4). It is possible, for instance, that the lack of correlation for the Silent Tapping conditions in most cases may reflect generally unreliable production rates for this task. We assessed reliability by averaging IOIs across the first trial for each item in a task (for Silent Tapping, this is just the mean produced rate for a single trial) and comparing this with the comparable mean for the final trial (trial 3 for production of Speech, Sung Melody, or Tapped Melody; trial 2 for Silent Tapping). All correlations were strong, positive, and statistically significant, indicating high reliability. High reliability between Silent Tapping trial 1 (before any auditory feedback trials) and trial 2 (in middle of study) indicates that any effects of auditory imagery did not significantly modulate the pattern of individual differences in tapping rate for this task. The plot in Figure 4D removes one participant from the Tapped Melody condition whose mean IOI for trial 1 (1.98) was highly discrepant from trial 3 (0.35), in contrast to other participants. When this participant was included, the correlation was much smaller but still significant at *p* < 0.05, *r*(56) = 0.25, *p* = 0.027.

**Figure 5:**
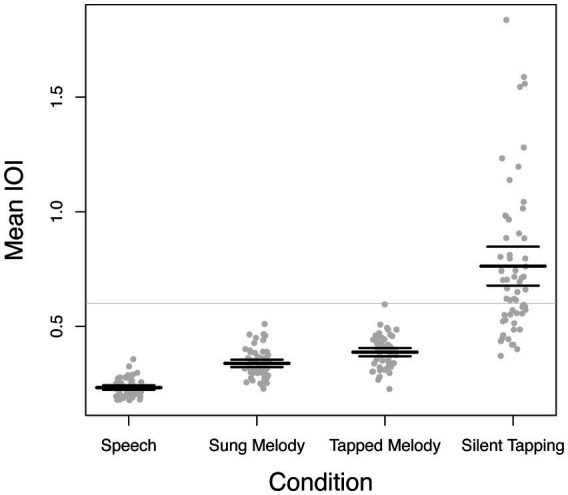
Mean production rate across tasks. Each dot indicates each participant’s mean IOI (in seconds) across all trials and items for a given task. The bold lines show means and upper and lower lines represent 95% confidence intervals for each task. *Y* axis units are in seconds per IOI.

Although our main focus is on correlations across tasks with respect to individual differences in SPR, we also analyzed mean SPR across tasks to assess whether the present data replicate results found in other studies. The mean IOI across all trials and items for each participant and task were analyzed via a within-participants single-factor ANOVA, which yielded a strong and significant effect of task, *F*(3, 171) = 113.43, *p* < 0.001, $\eta_{p}^{2}$ = 0.67. Means and distributions are shown in Figure 5. Post-hoc tests (Bonferroni corrected) revealed significant contrasts across all pairs of conditions. Mean IOIs thus proceeded in an ordered manner with Speech (*M* = 0.223, SE = 0.005) < Sung Melody (*M* = 0.339, SE = 0.008) < Tapped Melody (*M* = 0.387, SE = 0.009) < Silent Tapping (*M* = 0.763, SE = 0.042). This reflects what has been reported elsewhere (Coleman et al., 2024; Pfordresher et al., 2021). It is worth noting that none of the conditions approximates the 0.6 sec IOI rate first reported by Fraisse (1982), and often repeated in the literature (e.g., Honing, 2013), as shown by the light gray line. The correlations across tasks that we report thus were found in the context of mean differences in production rate that are typical for the tasks we used.

## Discussion

We used a set of tasks to assess whether individual differences in spontaneous production rates (SPRs) reflect shared timekeeping mechanisms based on the effector system used to generate rhythmic actions and/or the auditory feedback generated by those actions. We tested three hypotheses concerning the basis for the selection of an optimal production rate, observed via SPRs: basis on shared effector systems (H1), basis on the presence or absence of auditory feedback (H2), or basis on domain-specific differences in the structuring of information within a domain (H3). Table 4 provides a qualitative summary of how well the pattern of correlations across tasks fit these hypotheses, using both positive (significant correlation) and negative (nonsignificant correlation) evidence. Results clearly support the auditory feedback hypothesis over the other hypotheses. As shown in Table 4, all significant and non-significant correlations are consistent with this hypothesis, and with the results of Tranchant et al. (2022). By contrast, the pattern of results is only consistent with other hypotheses at rates suggesting chance performance.

**Table 4:** Qualitative assessment of support for hypotheses.

Correlation	Significant	Consistent with?		
		Effector (H1)	Feedback (H2)	Domain (H3)
Sung Melody/Speech	Y	✓	✓	✓
Sung/Tapped Melody	Y	✗	✓	✓
Speech/Tapped Melody	Y	✗	✓	✗
Sung Melody/Silent Tap	N	✓	✓	(n/a)
Tapped Melody /Silent Tap	N	✗	✓	(n/a)
Speech/Silent Tap	N	✓	✓	(n/a)
*N Support*		3/6	6/6	1/3

Each row reflects a correlation illustrated in Figure 2. Significance coded based on *p*-values from those correlations. Check marks indicate results that are consistent with a hypothesis, whereas *x*-marks indicate lack of support. Conditions include repetition of spoken phrases (Speech), singing familiar melodies on ‘da’ (Sung Melody), producing a melody on a keyboard by tapping a single key repeatedly (Tapped Melody), or tapping on a keyboard with no auditory feedback (Silent Tapping). H1: effector specificity, H2: auditory feedback presence, H3: domain specificity.

The primary finding of this experiment (H2) was that spontaneous rates vary across individuals in similar ways for tasks in which production generates auditory feedback (such as the SPR, spontaneous production rate), but not for tasks in which production does not generate auditory feedback (such as the SMT, spontaneous motor tempo). These findings are consistent with Tranchant et al. (2022) who speculated that differences in SPR and SMT values within individuals may be related to the fact that the auditory feedback present in SPR tasks provides error correction for timing. Another possibility is that SPR tasks are used to generate auditory patterns that may convey meaningful information to a perceiver. It is possible that the engagement of motor systems in tasks that contain a goal to create perceptual patterns may be less strongly associated with biomechanical constraints than tasks that do not contain perceptual goals.

The findings reported here differ from two other studies that we know of that reported positive correlations between SMT and SPR tasks. Engler et al. (2024) reported positive correlations between conditions similar to our Tapped Melody condition and silent tapping condition, and Pfordresher et al. (2021) reported a significant correlation between spontaneous rates of production of melodies on a piano and silent tapping. We suggest that these differences reflect sensitivity of SMT tasks to carryover effects from condition orders. Similar to what we reported in the present *Results* section, Pfordresher et al. (2021) found that the significant correlations across conditions varied depending on whether SMT was measured before or after the piano production task, with a significant correlation found for SPR and SMT only when SMT was measured after SPR. Although Engler et al. (2024) did not report such an analysis, they note that their SMT condition never occurred at the start of the session, but instead always occurred at a position intermingled with several other conditions (including SPR). Thus, we propose that other positive correlations between SMT and SPR may reflect a carryover of auditory imagery and/or motor imagery commonly seen in musicians (Highben and Palmer, 2004). An important implication of these results is that the use of SMT as a generalizable measure of internal timekeeping must be handled with care, given its potential vulnerability to order effects.

The current findings are also consistent with a timekeeping mechanism that is domain- and effector-general (contrary to H1 and H3), consistent with cognitive clock-counter models of timekeeping (e.g., Killeen and Weiss, 1987; Wing and Kristofferson, 1973). The correlation reported here between SPRs arising in Sung Melody and Speech production is consistent with the results of Coleman et al. (2024) and suggests domain-general timekeeping when sequences are produced with the same (vocal) effector system. Correlations of these tasks with the Tapped Melody condition suggest timekeeping that generalizes across effectors both within domain (Tapped with Sung Melody) and even across domains (Tapped Melody with Speech). The significant (although modest compared to others) correlation between Tapped Melody and Speech production was not consistent with the null (near zero) correlation between piano and speech production reported in Pfordresher et al. (2021) and deserves consideration. Some possible explanations for these different results include the use of familiar tunes that were associated with lyrics (used in the current study but not the previous study), the complexity of the fingered motor task (low in the current study—single-finger tapping—relative to the previous study), and degree of musical training (untrained participants in the current study, pianists in the previous study).

In sum, the present findings suggest that optimal rates may be defined by *the incorporation of sensorimotor plans to form action-sound contingencies*, based on the integration of motor actions with auditory feedback (cf. Tranchant et al., 2022). This conclusion is supported both by positive evidence (correlations across SPR tasks) as well as by Bayesian statistics that suggest support for the null hypothesis with respect to correlations across SPR and SMT tasks. The fact that music can be produced by different effector systems (including vocal and arm movements) makes this domain uniquely suitable for decoupling auditory from effector-based information.

## Data Availability

The raw data supporting the conclusions of this article will be made available by the authors without undue reservation.
